# Paternal grandmothers’ attitudes and beliefs toward newborn care practices in rural Ghana

**DOI:** 10.1186/s12887-026-07176-9

**Published:** 2026-06-25

**Authors:** Abdul-Mumin Amankwa, Florence Naab, Ernestina Asiedua, Deborah Armah, Mary Ani-Amponsah

**Affiliations:** 1 Department of Maternal and Child Health, School of Nursing and Midwifery, University of Ghana, Legon, Accra, Ghana; 2 Ministry of Health, College of Nursing and Midwifery, PO Box 10, Nalerigu, North East Region Ghana

**Keywords:** Traditional, Newborn care practices, Daboya, Grandmothers, Paternal, Qualitative study, Neonatal health

## Abstract

**Background:**

Newborn care practices, especially in low- and middle-income countries (LMICs) such as Ghana, are influenced by the traditional caregiving roles held by family members, particularly grandmothers. The Theory of Planned Behaviour (TPB) was used to explore and describe the attitudes and beliefs associated with newborn care practices among grandmothers in rural Ghana.

**Methods:**

An exploratory descriptive qualitative design was used in this study. Data were purposively collected through semi-structured interviews with 14 grandmothers in Daboya, Ghana, who cared for newborns. Data were analyzed using thematic analysis following Braun and Clarke’s six-phase method.

**Results:**

The study explored two key themes: grandmothers’ attitudes and societal norms towards newborn care practices. A mix of positive, negative, and uncertain attitudes was found among the grandmothers. Many confidently used herbal remedies and immediate bathing based on personal experience, while some rejected harmful practices such as tribal marking and force-feeding. Others combine home care with modern medicine. Strong beliefs about colostrum, pre-lacteal feeds, and infant seclusion, along with a focus on spirituality in protection and naming, reflect the influence of traditional norms and family customs that have been passed down through generations.

**Conclusion:**

This study explored grandmothers’ attitudes toward newborn care, revealing diverse views. Traditional norms and family customs have significantly influenced newborn care practices across generations. Future research should examine the influence of grandparents on newborn care to promote effective practices and safely blend them with modern healthcare for better outcomes.

**Supplementary Information:**

The online version contains supplementary material available at https://doi.org/10.1186/s12887-026-07176-9.

## Background

Globally, newborn care practices include a range of interventions aimed at promoting survival and development of newborns. These include establishing maternal-infant bonding, supporting physiological adaptation to life outside the womb, and implementing preventative healthcare measures [1, 2]. Routine practices, such as monitoring vital signs, regulating body temperature, and conducting newborn screenings, must be balanced with efforts to minimize unnecessary or invasive procedures [3]. Despite the existence of evidence-based guidelines, several traditional practices remain widespread, particularly in rural communities. Practices such as delayed breastfeeding initiation, early newborn bathing, application of substances to the umbilical cord, and discarding colostrum are still common and have been associated with increased neonatal morbidity and mortality [4].

Grandmothers are widely acknowledged as central figures in the transmission of cultural norms and health practices within the family. They are recognized as custodians of family health and tradition, especially in non-Western societies, where elders play an essential role in intergenerational knowledge transfer [5]. In many households, particularly in low-resource settings, newborn care is provided within the home environment. Grandmothers, along with other family members, often serve as key caregivers, providing support to mothers and making critical decisions related to newborn care. Comparative reviews across Asia, Africa, Latin America, and Indigenous communities affirm the consistent involvement of grandmothers in maternal and child health practices, often shaped by local traditions and beliefs [6, 7].

The influence of grandmothers on newborn care practices is reflected in various cultural contexts. A study in India identified positive maternal behaviors, such as exclusive breastfeeding for six months, timely initiation of the first bath, and appropriate health-seeking behaviors, many of which were guided or supported by elderly women [8]. Conversely, in Nigeria, evidence suggests that childbirth is often attended by either a traditional birth attendant or the grandmother, with newborns bathed within 30–45 min following delivery [9]. This practice diverges from the recommended neonatal guidelines.

The postpartum period often presents significant challenges to new mothers, including physical exhaustion, underlying health issues, multiple births, and complications from cesarean sections. In such contexts, family support is indispensable. Grandmothers and other caregivers, such as traditional birth attendants, frequently step in to provide care that mothers may be temporarily unable to give [2, 10]. While grandmaternal involvement in newborn care is a global phenomenon, its manifestations vary across regions.

In the United States, grandparents often provide childcare with a sense of honor in caring for their grandchildren [11]. Similarly, in Chinese communities, grandparents may travel abroad or host their daughters during the postnatal period to provide culturally prescribed care [12]. In African contexts, such as Kenya, maternal grandmothers are influential advocates of traditional breastfeeding and childcare practices [13].

Ensuring that every child receives essential newborn care is a global imperative, as recognized by the Convention on the Rights of the Child [14]. The newborn period is a critical window for health and developmental outcome. However, financial constraints, limited access to healthcare, and entrenched social norms often hinder optimal newborn care in many low- and middle-income countries [15, 16]. To address these gaps, several countries have implemented community-level strategies, such as Ghana’s Community-based Health Planning and Services (CHPS), which aim to expand access to primary care and improve maternal and newborn health outcomes [17].

Despite these efforts, rural regions of Ghana, including areas such as Daboya in the North Gonja District, continue to face structural barriers. Limited healthcare infrastructure, shortages of skilled health personnel, and logistical challenges impede the full realization of evidence-based care for newborns [18]. In these settings, newborn care practices are predominantly guided by paternal grandmothers based on their cultural beliefs and family traditions. These practices are often supervised or performed by paternal grandmothers, whose role becomes even more critical in the absence of formal healthcare support [19].

Daboya is the capital of the North Gonja District and the traditional seat of the Wasipe traditional area. Daboya as a community comprises different clans and traditional gates, which makes it unique in its indigenous traditional beliefs and practices. The people in Daboya practice extended and patriarchal family systems, and as such, new mothers live in the same homes with their in-laws. It is a tradition in the community for the new mother and her baby to move from her husband’s room or house into her mother-in-law’s room (the baby’s paternal grandmother) for supportive care of both the mother and the newborn. These paternal grandmothers usually have absolute control over the care of their grandchildren, based on their experiential knowledge, even when the parents of the baby are against any practice. These paternal grandmothers are regarded as custodians of traditional newborn care practices and can provide the information needed for this study.

In light of this, this study aimed to explore the attitudes and beliefs of paternal grandmothers regarding newborn care practices in a rural Ghanaian community using the Theory of Planned Behaviour (TPB) as the framework. This study employed some constructs of the Theory of Planned Behaviour (Attitudes and Subjective norms) as its theoretical framework, drawing on Ajzen [20] to explore and describe paternal grandmothers’ attitudes and beliefs towards newborn care in rural Ghana. Using this framework allowed the study to capture both attitudes and beliefs within families and communities, aligning with the aims of this study. The findings aim to inform interventions that strengthen community health systems, improve caregiver training, and align local newborn care practices with global standards for neonatal care. Ultimately, this study contributes to the broader objective of achieving Sustainable Development Goal 3: ensuring healthy lives and promoting well-being for all.

## Materials and methods

### Research design

This study employed an exploratory descriptive qualitative design [21] to explore the attitudes and beliefs associated with newborn care practices among paternal grandmothers in Daboya, Ghana. This design is considered most appropriate for a qualitative study because it enables researchers to contextualize newborn care practices from a broader perspective of participants’ natural settings [22].

### Sampling technique and sample size

The researchers purposively recruited 14 paternal grandmothers due to their direct involvement in caring for the newborns and living with new mothers.

The inclusion criteria were as follows: being the mother or father of an infant (the baby’s paternal grandmother), residing in the community, and caring for an infant (≤ 6 months old) within the past year. This was because they could provide the information needed for the study. Traditionally, new mothers stayed with their husbands’ mothers for some months immediately after delivery to receive support in caring for their babies. Village health volunteers and a local health clinic helped identify eligible paternal grandmothers in the study. Data saturation was reached with the 14th participant when no new information emerged, and recruitment was concluded.

### Data collection procedures

Data were collected by two experienced researchers fluent in Gonja (the local language). The researchers developed a semi-structured interview guide based on the literature and TPB constructs (Supplementary 1). After obtaining informed consent, interviews were conducted at the participants’ homes. Each session lasted 30–60 min and was audio-recorded with permission. The interviews were conducted in the Gonja language because the participants felt comfortable communicating in this language. The interviewers also noted nonverbal gestures in a field diary, which supported the interviews during data analysis. To ensure confidentiality, the participants’ real names were replaced with pseudonyms to maintain privacy and anonymity. The questions explored care practices (e.g., feeding, bathing, cord care), beliefs about their benefits or harms, and influences from family and tradition. The interviews were transcribed verbatim in Gonja and then translated into English by the research team. A second bilingual researcher reviewed the translations to ensure that the meanings were preserved (back-to-back translation).

### Data analysis

Data were analyzed manually using thematic analysis [23] following Braun and Clarke’s six-phase method [24]. First, the researchers read all the transcripts repeatedly for familiarization. Next, they generated initial codes inductively, staying close to the data. The codes were collated into candidate themes, which were iteratively reviewed and refined. The final themes and sub-themes were defined by consensus among the researchers. Finally, the data were organized into a report with deep descriptions supported by exemplar quotes for each theme to illustrate paternal grandmothers’ perspectives.

## Results

### Participants’ background

All participants were speakers of the Gonja language, with ages ranging from 44 to 75 years. Among the fourteen participants, twelve had no formal education, while one had completed primary school. Only one participant reached the form three level of education, representing the highest educational attainment in the group. Thirteen participants identified as Muslims, whereas one identified as a Christian. Regarding their marital status, nine participants were married, whereas five were widows. All participants had experienced caring for newborns. Twelve participants had single newborn grandbabies, while two had a pair of twins each. Table 1 summarizes the demographic characteristics of the 14 mothers-in-law who participated in this study.

**Table 1:** Demographic characteristics of study participants (*N* = 14)

Pseudonyms	Age	Educational background	Religion	Marital status	Language
PGM1	50 years	No formal Education	Muslim	Widow	Gonja
PGM2	60 years	No formal Education	Muslim	Widow	Gonja
PGM3	51 years	No formal Education	Muslim	Married	Gonja
PGM4	45	No formal Education	Muslim	married	Gonja
PGM5	44 years	No formal Education	Muslim	married	Gonja
PGM6	53 years	No formal Education	Muslim	married	Gonja
PGM7	48 years	No formal Education	Muslim	married	Gonja
PGM8	75 years	No formal Education	Muslim	married	Gonja
PGM9	50 years	Form three level	Christian	Widow	Gonja
PGM10	58 years	No formal Education	Muslim	Married	Gonja
PGM11	51 years	Primary level	Muslim	Married	Gonja
PGM12	45 years	No formal Education	Muslim	married	Gonja
PGM13	69 years	No formal Education	Muslim	Widow	Gonja
PGM14	50 years	No formal Education	Muslim	Widow	Gonja

### Themes and sub-themes

Two main themes, guided by the Theory of Planned Behaviour (TPB), were used to generate the data, as shown in Table 2.

**Table 2:** Themes and subthemes

Themes	Subthemes
Attitudes of Paternal grandmothers towards newborn care practices	a. Positive appraisal b. Negative appraisal c. Uncertainty about newborn care
Societal Norms of Paternal grandmothers about newborn care practices	a. Beliefs b. Spirituality c. Traditional norms

#### Theme 1: attitudes of paternal grandmothers towards newborn care practices

The first major theme identified the attitudes of paternal grandmothers. Some paternal grandmothers appraised some practices as good, while others were appraised as bad. They engaged in newborn care practices that they appraised positively for their grandchildren and disapproved of those practices that they appraised negatively. Positive and negative appraisals and uncertainty about newborn care were the categories of attitudes reported.

### Positive appraisal towards newborn care practices

Most participants expressed a positive perspective on newborn care practices. Many felt confident in caring for their newborn grandchildren because of prior experience. They utilized herbal concoctions to treat newborn ailments and promote strength and health. In addition, they believed that early bathing of the newborn helped remove vernix and blood, thereby preventing body odor.

Here are some comments from PGM6 and PGM7:

“I have taken care of children before this one, and when I applied these herbs to those children within some few days the tribal marks wounds were gone. So, I realized it was good to mix the oil and the herbs on my grandchild’s wounds. This will heal the wounds faster if I apply them” PGM6.

“I boil neem tree leaves for the baby to drink to treat the fever and malaria in him. Last week when this baby’s body became warm, it was those leaves I plugged outside and boiled and bathed him and the temperature reduced” PGM7.

Also, PGM2 and PGM5 comments reflect these appraisals:

“The last time we had a child delivered at home by a Traditional Birth Attendant (TBA), I quickly bathed the child to remove those white substances and blood on the body” said PGM2.

“Newborn babies come out with some white substances and blood on their bodies, if you do not remove them, the baby develops body odour. Immediate bathing would prevent that” PGM5.

### Negative appraisal towards newborn care practices

Some participants viewed specific newborn care practices negatively based on their prior experiences. They chose not to engage in these practices, citing the potential harm. Disapproval focused on tribal markings, herbal concoctions, and force-feeding infants. Some participants shared these concerns:

“Some of the practices we have at home are not good for the babies. I have refused to give this baby tribal marks because I do not want to inflict too much pain on the baby. These tribal marks have denied a lot of people opportunities because, their tribe is easily identified” PGM7.

“I would not be able to care for the baby with tribal marks very well. If I apply shea butter to the marks and umbilical cord, the child’s wound will be delayed in healing, which would be bad. This is why I did not allow the baby to be given the tribal marks” PGM3.

PGM10 had this to share:

“I have not bathed the baby in any herbs or given herbal concoction to drink. The herbs we bath these babies changes the skin colour to dark. Also, the way we give the herbs to newborn babies is not good, we force them to drink them, which sometimes chokes them.” PGM10.

### Uncertainty about newborn care

Some paternal grandmothers expressed uncertainty, neither fully supporting nor rejecting the traditional newborn care. They favored integrating home-based care and medical care approaches, encouraging health facility visits when needed: Here are some of their comments:

“The TBA will ask the daughter-in-law to bring the items required to perform the rituals and she will perform the rituals for the nipples to open for more milk to flow. This does not prevent us from sending the baby to the clinic for the healthcare workers to help in checking his health status” PGM6.

“Although my son always looks for some herbs that are boiled and the concoction is used to bathe the baby. Sometimes it is the TBA who gives me some herbal concoctions, and if it is a sickness that deserves to be sent to the hospital, then, I do not hesitate to do so” PGM14.

#### Theme 2: societal norms of paternal grandmothers about newborn care practices

The second significant theme identified in this study was the societal norms of paternal grandmothers regarding newborn care practices. The majority of participants expressed a wide range of societal norms that influence newborn care practices, which were categorized into three sub-themes: beliefs, spirituality, and traditional norms.

### Beliefs about newborn care

Nearly all participants held beliefs about caring for their newborn grandchildren. Some paternal grandmothers thought colostrum was “bad milk” and should be discarded. Paternal grandmothers provided pre-lacteal feeds to babies, symbolizing a connection to their ancestors. Additionally, allowing newborns outside before 40 days of age and nighttime exposure was viewed as taboo. The participants expressed diverse perspectives on these practices.

PGM10 and PGM13 believed that:

“The first breast milk from a mother is expelled and thrown away to prevent the baby from sucking it. It is a “bad milk” and the child must not be allowed to drink it” PGM10.

“Immediately the baby comes out and is cleaned, she is sent before the oracles and incantations are done for our ancestors to bless the cow milk. I then drop some of the milk into the baby’s mouth. If it is a boy, it would be 3 drops and if a girl then 4 drops to signify that the baby dinned with the ancestors first before any other food.” PGM13.

PGM1 and PGM8 had the following to say about seclusion:

“I still maintain the belief that until my grandchild is 40 days old, he would not come outside the room. My children went through this, and nothing happened to them.” PGM1.

“it is a belief that, the newborn who is not up to 40 days old is not allowed to watch the skies, he might be possessed by the evil spirits. Even when the baby gets to the 40th day, some rituals are performed and even after the 40th day when the baby is now allowed to come outside the room, he is sent back when it is getting dark” PGM8.

### Spirituality about newborn care

The second sub-theme identified within this theme was the role of spirituality in newborn care practices. Some paternal grandmothers believed that spiritual consultations and rituals were essential for caring for their newborn grandchildren. Additionally, some participants mentioned introducing their grandchildren to family deities to ensure protection from spiritual harm. Others held the belief that naming their newborns after ancestors or shrines would provide spiritual safeguarding.

PGM3 and PGM12 shared the following insights:

“If the child is crying and wants a name, animals are killed to appease the ancestors for a name of an ancestor to be given to him. This child was named after his great grandfather, and that is why I call him my grandfather. Some children come from the shrines and they are named after those shrines” PGM3.

“A red cock was killed to appease the ancestors on the day of the naming ceremony and the blood from the cock was used on his forehead to signify acceptance from the ancestors” PGM12.

Also, PGM14 expressed this on protection:

“In our tradition, I tied a thread with cowries on my grandchild hand and waist after some number of months after birth and my reason is to spiritually prevent any evil spirits from coming into contact with my grandchild” PGM14.

### Traditional norms about newborn care

A prevailing belief among nearly all participants was the importance of traditional norms. Some paternal grandmothers engaged in practices widely accepted within their families and communities for newborn care. They preferred to uphold the customs passed down from their parents and elders rather than adopt medical care approaches for newborn care. Some participants felt that adhering to family traditions set them apart from other families.

As expressed by PGM8:

“Actually, in this house, we believe in the ‘k’kpante’ (crab shell). This would be put in the fire and would be used to touch the baby’s lips. This is believed to help newborns become truthful and eloquent as they grow. This has been the practice of our forefathers for a long time,” PGM8.

Also, PGM10 and PGM12 shared their views:

“Actually, this is what we all went through with our parents, and that was applied to my children, and now I am applying the same to my grandchildren too. They will one day apply it to their children. These practices are family norms in the community. When I got married to this family, I met my mother-in-law who told me all what it takes to take care of a newborn and what not to do…” PGM10.

“That was what my parents met in the family and passed it on to me, so I must also do the same to maintain the norm, not exactly what they will want me to do, but what is accepted in the family by all members is what is necessary”, PGM12.

## Discussion

The study explored and described the attitudes and beliefs associated with newborn care practices among paternal grandmothers in rural Ghana. This study was underpinned by the Theory of Planned Behaviour (TPB) to explore and describe the attitudes and beliefs of paternal grandmothers. The findings reveal that paternal grandmothers play a crucial role in decision-making regarding home-based newborn care, influenced by deeply entrenched cultural norms and spiritual interpretations of health. These beliefs persist not solely due to a lack of knowledge but are also reinforced by social expectations, symbolic meanings, and the moral responsibility that grandmothers hold within the patrilineal family structure. Within the framework of the Theory of Planned Behaviour (TPB), many of these practices exhibit strongly favourable attitudes toward traditional care and powerful subjective norms that promote conformity to community expectations.

It is worth noting that paternal grandmothers in this study endorsed herbal baths, shea butter for the umbilical cord, and herbal concoctions for treating newborn ailments. These practices align with earlier findings from Nigeria, where herbal remedies and local substances like shea butter, oils, and lotions were used on newborn babies, especially on their umbilical cords [9, 25]. In some instances, the use of these local practices has shown perceived benefits. Studies have documented cases in which herbal applications helped maintain umbilical cord integrity and were believed to reduce jaundice-related bilirubin levels in newborns [26]. However, other studies have raised significant concerns regarding this approach. Some of these herbal treatments have serious or fatal consequences for newborns, particularly when neonates are forced to ingest potentially harmful concoctions [27]. These practices may interfere with the healthy growth and development of newborns. The practice of herbal cord care and medicinal concoctions can be analyzed through attitudes. Paternal grandmothers exude confidence in their knowledge, reinforcing their intention to maintain these traditions. Their authoritative role legitimizes these practices, especially when younger mothers have limited decision-making autonomy. In patrilineal communities, social structures elevate grandmothers as custodians of tradition and primary advisors in newborn care. Nurses and midwives in postnatal and community settings should involve paternal grandmothers in health education sessions, alongside mothers. Community durbars and antenatal classes can include discussions on safe cord care, blending essential newborn care explanations with respect for traditional knowledge. Engaging community leaders to support safer alternatives can help promote evidence-based practices.

The findings of this study also revealed a widespread endorsement by paternal grandmothers of immediate postnatal bathing of newborns. This practice was often rationalized as a means of preventing body odour by removing the vernix and blood stains. Similar practices have been reported in previous studies, where early bathing of newborns was customary, particularly after vaginal deliveries, to remove birth-related residues and prevent newborn skin infections [28]. In Ethiopia, early bathing of newborns is reported by 55% of postpartum women, a trend attributed in part to the low educational levels of caregivers [29]. In contrast, studies from different settings supported postponing the initial bath until after the newborn achieves 6 to 24 h post-delivery, in order to prevent hypothermia and other complications [30, 31]. Early bathing aligns with positive attitudes towards cleanliness and social norms, where visible birth residues are seen as impure. This belief endures as cleanliness is linked to dignity and proper motherhood, with deviations inviting social criticism. Postnatal counselling should explain the protective role of vernix and the risks of hypothermia in simple terms. Demonstrations during facility deliveries and follow-up home visits by community health nurses can promote delayed bathing practices while respecting families’ cleanliness rituals.

In addition, a significant subset of paternal grandmothers in this study expressed uncertainty regarding the exclusive reliance on home-based care. These paternal grandmothers indicated a willingness to seek medical assistance when traditional care methods were ineffective. This uncertainty reflects a significant decrease in optimal newborn care practices, where informal caregivers struggle to navigate between seeking home-based care practices and medical healthcare practices for their newborns [16, 32]. These findings suggest that grandmothers may perceive medical care as a secondary option, pursued only when local newborn care practices fail. This finding illustrates a negotiation between traditional beliefs and changing norms influenced by increased access to medical services. While grandmothers trust traditional practices, their willingness to seek facility care suggests a shift in intentions due to greater engagement with the healthcare system. Health systems should strengthen respectful maternity care and community outreach to build trust with grandmothers. Rather than positioning medical care as a replacement for tradition, nurses and midwives could frame facility care as complementary, thereby gradually influencing norms without undermining grandmother authority.

Another interesting finding of this study pertains to the strong spirituality and cultural beliefs expressed by paternal grandmothers concerning newborn care. Some paternal grandmothers identified colostrum as “bad milk”, leading to its immediate discarding after birth. This belief is consistent with other studies that attribute colostrum avoidance to cultural misconceptions [4, 33]. Conversely, research has indicated that caregivers who understand and recognize the health benefits of colostrum as an essential nutrient are more likely to support its use [34, 35]. Discarding colostrum deprives newborns of vital antibodies, thereby increasing their susceptibility to infection. Colostrum avoidance stems from deeply rooted subjective norms passed down through generations. The labeling of colostrum as “bad milk” is based on symbolic interpretations of its color and texture, rather than scientific reasoning. Grandmothers, seen as sources of wisdom, significantly influence maternal intentions and early feeding practices. Therefore, breastfeeding promotion should specifically target grandmothers in antenatal and postnatal education. Visual demonstrations and testimonials from respected local women, along with involvement from community leaders, can help change norms about colostrum use.

The study also reported that newborns in the community were often welcomed with pre-lacteal feeds such as cow’s milk or millet flour, practices that are deeply rooted in custom. This finding corresponds with studies in other contexts where honey and other substances are provided to newborns as part of religious or cultural rituals [36–38]. Although culturally significant, these pre-lacteal feeding practices can interfere with the early initiation of breastfeeding, suppress maternal lactation, and expose infants to gastrointestinal infections [39]. Pre-lacteal feeding continues because it represents social acceptance and ancestral ties, reinforcing strong norms. Grandmothers’ influence on feeding decisions further solidifies this practice, especially in extended families where mothers may have less negotiating power. Community health campaigns should position early breastfeeding initiation as both medically beneficial and culturally commendable. Collaborating with traditional and religious leaders can help reinterpret welcoming rituals to support exclusive breastfeeding.

A further observation in this study was the practice of seclusion, whereby newborns were kept indoors for 40 days, as a protective spiritual measure. This tradition, upheld by some paternal grandmothers, reflects similar cultural norms observed in countries such as Iraq and the Philippines, where postpartum seclusion is a deeply embedded practice [40]. Conversely, research conducted in the United States highlights potential developmental concerns related to maternal-infant separation, particularly its impact on early bonding and timely access to healthcare [41]. Seclusion practices reflect how social structures and spiritual beliefs influence caregiving. In patrilineal systems, paternal grandmothers enforce these norms through moral authority. Although seclusion aims to be protective, rigid expectations can hinder timely health-seeking behavior. Structured home visitation programs by community health nurses can provide newborn assessments, immunization tracking, and breastfeeding support while respecting cultural seclusion practices. Collaborating with community leaders can help make these visits culturally accepted extensions of care.

The current study also revealed the spiritual and traditional norms of newborn care by paternal grandmothers. Practices such as naming rituals involving ancestral appeasement, the tying of protective amulets, and sacrificial rites reflect the deeply held belief that spiritual forces influence infants’ health and well-being. Similar findings have been reported in parts of Africa and Indonesia, where rituals are performed to invoke ancestral protection and ward off evil spirits [42, 43]. These beliefs are integral to the community’s worldview and influence caregiving decisions from birth onward.

While such practices may seem incongruent with medical care, they offer emotional reassurance to caregivers and are deeply rooted in cultural cosmologies. Studies have shown that disregarding these norms may alienate caregivers and reduce trust in formal health systems [32]. In contrast, inclusive health education that respects spiritual beliefs can foster greater community collaboration and improve neonatal outcomes [7]. Naming practices involving ancestors and rituals for spiritual protection may serve symbolic roles that build familial bonds and social recognition for newborns [8]. From a theoretical standpoint, these practices illustrate the interaction between attitudes and subjective norms. Understanding these dimensions is essential for culturally responsive health interventions. Health initiatives must adopt a culturally sensitive approach that integrates traditional beliefs into maternal and child health programs, promoting respect and participation. Strategies like routine home visits, culturally tailored education, and partnerships with traditional leaders can effectively connect spirituality with evidence-based newborn care.

### Strengths and limitations

This study explored newborn care practices among paternal grandmothers, emphasizing their significant role in these practices. It advocates for family centered care that combines traditional knowledge with evidence-based practices and encourages community programs to support families.

However, this study primarily focused on paternal grandmothers, excluding the views of mothers and fathers on newborn care. Additionally, the translation of some indigenous words posed a challenge that may have altered their meaning despite attempts at back-translation. Potential bias due to conducting interviews in the participants’ homes.

## Conclusion

Acknowledging the role of paternal grandmothers in neonatal care is essential for community health in rural Ghana. The use of the Theory of Planned Behavior (TPB) as the framework allowed the study to capture both attitudes and beliefs within families and communities, aligning with the aims of this study. Paternal grandmothers’ cultural beliefs heavily influence the newborn care practices. Engaging paternal grandmothers through respectful conversations can help integrate evidence-based newborn care while honoring family traditions. Future research should examine the influence of grandparents on newborn care to promote effective practices.

## Supplementary Information

Supplementary Material 1.

## Data Availability

The corresponding author will make the data used in this study available upon request.

## Abbreviations

NCP: Newborn care practices
WHO: World Health Organization
TPB: Theory of Planned Behaviour
PGM: Paternal Grandmother
