# Supplementary material for: Paternal grandmothers’ attitudes and beliefs toward newborn care practices in rural Ghana
Source: BMC Pediatr. 2026 Jun 25;26:900. doi: 10.1186/s12887-026-07176-9 (PMC13602496; doi:10.1186/s12887-026-07176-9)
Supplement: Supplementary file 1 [file 12887_2026_7176_MOESM1_ESM.pdf]

INTERVIEW GUIDE

Research Title: Planned behaviour of mother in-laws about traditional newborn care practices in the North Gonja District.

Principal investigator: Amankwa Abdul-Mumin

Address: School of Nursing and Midwifery, College of Health Sciences, University of Ghana, Legon.

Tel: 0247072482

SECTION A: Demographic Data

Code No./ Pseudonym:

Age:

Educational Level:

Religion:

Marital Status:

- Single ( )
- Married ( )
- Widow ( )

SECTION B: The questions are asked based on the constructs of the THEORY OF PLANNED BEHAVIOUR

Attitude towards the behaviour

1. How old is your grandchild?
2. How do you plan to care for your grandchild?
3. How long did it take you to care for your grandchild after birth? Why that long?
4. What do you think is the proper way of caring for a child below 6 months?
5. Do you involve your daughter in-law in the care of your grandchild?

Subjective Norm

1. What are some of the beliefs you have about caring for your grandchild?

2. Do you feel like you are being influenced by others to care for the newborn the way you are doing now?
3. Who is influencing you?
4. Why do you think those people want you to care for your grandchild that way?
5. Why will you practice what they tell you do?
6. What will happen if you do not care for the newborn the way you are being influenced to do it?

Perceived behavioural control

1. What makes it easier for you to practice traditional newborn care practices?
2. What makes it difficult for you to practice traditional newborn care practices?
3. What makes you think you are the best person to care for your grandchild?

Behavioural intention

1. How did you plan to take care of your grandchild?
2. What exactly was your plan towards the care of your grandchild?
3. Has that plan changed? If yes, why? And if no, why?
4. If the mother of your grandchild was not there, how will you care for the newborn?

Behaviour

1. How do you care for your grandchild now?
2. What are some of the substances that you give or are still giving to your grandchild?
3. Any reason for giving your grandchild those substances?
4. Where do you get newborn care practices information from?
5. Tell me what the nurse/midwife taught you about the care of your grandchild?
6. Explain how you care for your grandchild according to the way the nurses/midwives taught you?
